# Syntheses and structures of bis(2-amino­py­rimi­dine-κ*N*^1^)di­chlorido­zinc(II) (ortho­rhom­bic polymorph) and bis(2-am­ino­py­rimi­dine-κ*N*^1^)di­iodido­zinc(II)

**DOI:** 10.1107/S2056989026001878

**Published:** 2026-02-24

**Authors:** Christian Näther, Gaurav Bhosekar

**Affiliations:** a Institut für Anorganische Chemie, Universität Kiel, Max-Eyth.-Str. 2, 24118 Kiel, Germany; b Suman Ramesh Tulsiani Technical Campus - Faculty of Engineering. Kamshet, Pune, India; University of Aberdeen, United Kingdom

**Keywords:** crystal structure, coordination compounds, discrete complexes, hydrogen bonding, synthesis, zinc halide, 2-amino­pyrimidine

## Abstract

The title compounds, [ZnCl_2_(C_4_H_5_N_3_)_2_] (**1**) and [ZnI_2_(C_4_H_5_N_3_)_2_] (**2**) (C_4_H_5_N_3_ = 2-amino­pyrimidine), were prepared from a solvent mixture of tri­chloro­methane and methanol and structurally characterized. The asymmetric unit of **1** consists of one Zn^II^ cation that is situated on a twofold rotation axis and one chloride anion as well as one 2-amino­pyrimidine ligand in general positions. The asymmetric unit of **2** is built up of one Zn^II^ cation, two iodide anions and two 2-amino­pyrimidine ligands, all of them located in general positions. In both compounds, the metal cations are tetra­hedrally coordinated by two halide anions and two 2-amino­pyrimidine ligands. In compound **1**, the discrete complexes are linked by N—H⋯Cl hydrogen bonds into layers that are further connected by weak C—H⋯Cl inter­actions. In compound **2**, the complexes are connected *via* N—H⋯I hydrogen bonds into layers that are further linked by a number of N—H⋯I and C—H⋯I hydrogen bonds. Compound **1** represents a second polymorphic modification of ZnCl_2_(C_4_H_5_N_3_)_2_ that crystallizes in the ortho­rhom­bic non-centrosymmetric space group *Pba*2, whereas the known form crystallizes in the monoclinic, centrosymmetric space group *C*2/*c* [Lin & Zeng (2007 ▸). *Acta Cryst.* E**63**, m1597].

## Abstract 2

The crystal structures of ZnCl_2_(C_4_H_5_N_3_)_2_ (second polymorph) and ZnI_2_(C_4_H_5_N_3_)_2_ were determined. Both are built up from discrete tetra­hedral complexes that are connected *via* inter­molecular hydrogen bonds.

## 1. Chemical context

Investigations on the synthesis and crystal structures of new coordination compounds is still a major field in inorganic chemistry. Our inter­est is focused on coordination compounds based on transition-metal halides and N-donor coligands. In the beginning, we concentrated on compounds based on univalent cations such as Cu^I^ because they show a versatile structural behavior that can be traced back to the fact that the metal cations are frequently linked by bridging halide anions (Kromp & Sheldrick, 1999 ▸; Peng *et al.*, 2010 ▸; Näther *et al.*, 2001 ▸, 2002 ▸). Moreover, multiple compounds with a different ratio between the metal halide and the coligand are often observed (Näther & Jess, 2002 ▸; Näther *et al.*, 2003 ▸). In further work, we investigated coordination compounds with twofold positively charged metal cations such as Zn^II^ or Cd^II^, but compared to the copper(I) halides these show a limited structural variability (Bhosekar *et al.*, 2006 ▸; Neumann *et al.*, 2018*a* ▸,*b* ▸). Such compounds, however, are of inter­est because of their luminescence properties (Zeng *et al.*, 2010 ▸; Neumann *et al.*, 2018*a* ▸,*b* ▸; Jess *et al.*, 2020 ▸; Kokina *et al.*, 2020 ▸).

In the course of our systematic investigations, we became inter­ested in 2-amino­pyrimidine (C_4_H_5_N_3_) as coligand. On the one hand, the coordination of a metal cation might be more difficult, because of steric inter­ference from the neighbouring amino group, but on the other strong hydrogen bonding can be expected.

In this context it is noted that two compounds with the composition ZnCl_2_(C_4_H_5_N_3_)_2_ (CSD refcode YIDPAD; Lin & Zeng, 2007 ▸) and ZnBr_2_(C_4_H_5_N_3_)_2_ (LOBPOI; Qu *et al.*, 2008 ▸) are already reported in the CSD (Version 5.43, 2025; Groom *et al.*, 2016 ▸), as found using a CONQUEST (Bruno *et al.*, 2002 ▸) search. Both of them consist of discrete neutral complexes that are linked by inter­molecular hydrogen bonds. In the course of our investigations, we obtained crystals of the missing compound with ZnI_2_, and with ZnCl_2_ we obtained crystals of a second polymorph of ZnCl_2_(C_4_H_5_N_3_)_2_ (Lin & Zeng, 2007 ▸).
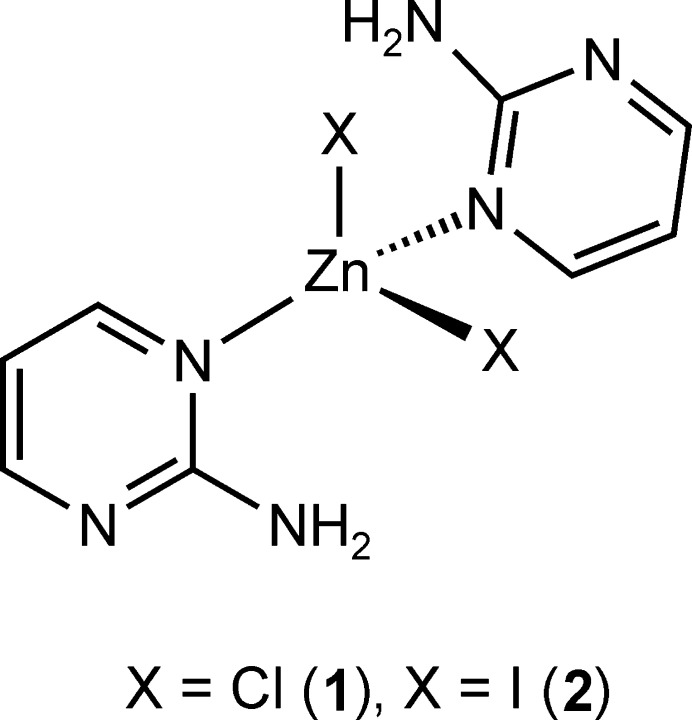


## 2. Structural commentary

**Figure 1:**
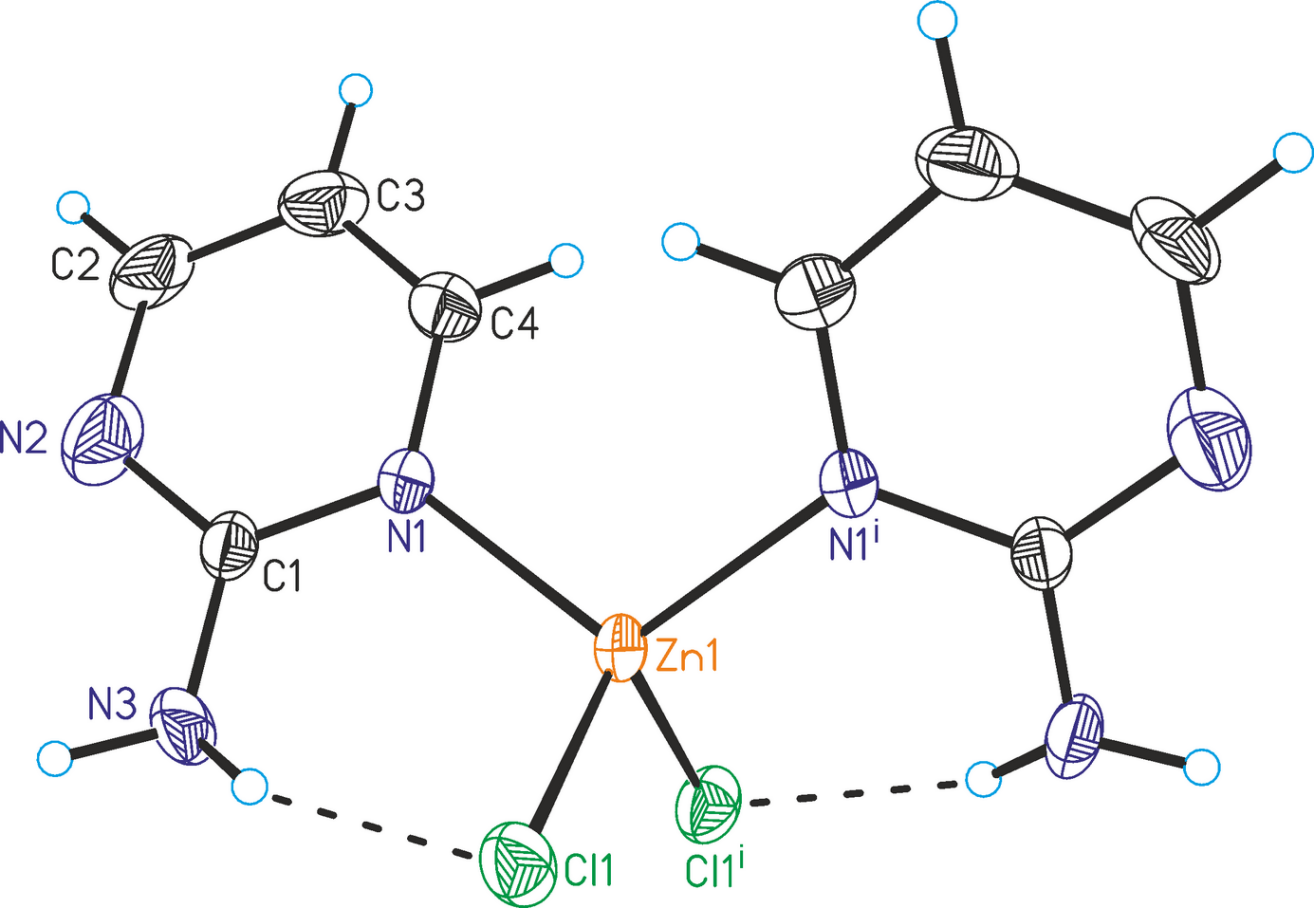
The mol­ecular structure of **1** with displacement ellipsoids drawn at the 50% probability level and intra­molecular N—H⋯Cl hydrogen bonding shown as dashed lines. Symmetry code: (i) −*x* + 1, −*y* + 1, *z*.

ZnCl_2_(C_4_H_5_N_3_)_2_ (**1**) represents a second polymorphic modification of the form that is already reported in the literature (Lin & Zeng, 2007 ▸). In contrast to the reported form that crystallizes in the centrosymmetric monoclinic space group *C*2/*c*, compound **1** crystallizes in the non-centrosymmetric ortho­rhom­bic space group *Pba*2. The asymmetric unit of **1** consists of one Zn^II^ cation located on a twofold rotation axis and one chloride anion and one 2-amino­pyrimidine ligand in general positions (Fig. 1 ▸). In the crystal structure, the Zn^II^ cations are fourfold coordinated by two symmetry-related halide anions and two symmetry-related 2-amino­pyrimidine ligands (Fig. 1 ▸). Bond lengths and angles show that the tetra­hedra are only slightly distorted (Table 1 ▸). The overall geometry is very similar to the form reported in the literature (Lin & Zeng, 2007 ▸). Compound **1** was crystallized from the mixed solvents of methanol and tri­chloro­methane (see *Synthesis and crystallization*) whereas the form reported by Lin & Zeng was crystallized from an ethanol solution.

**Figure 2:**
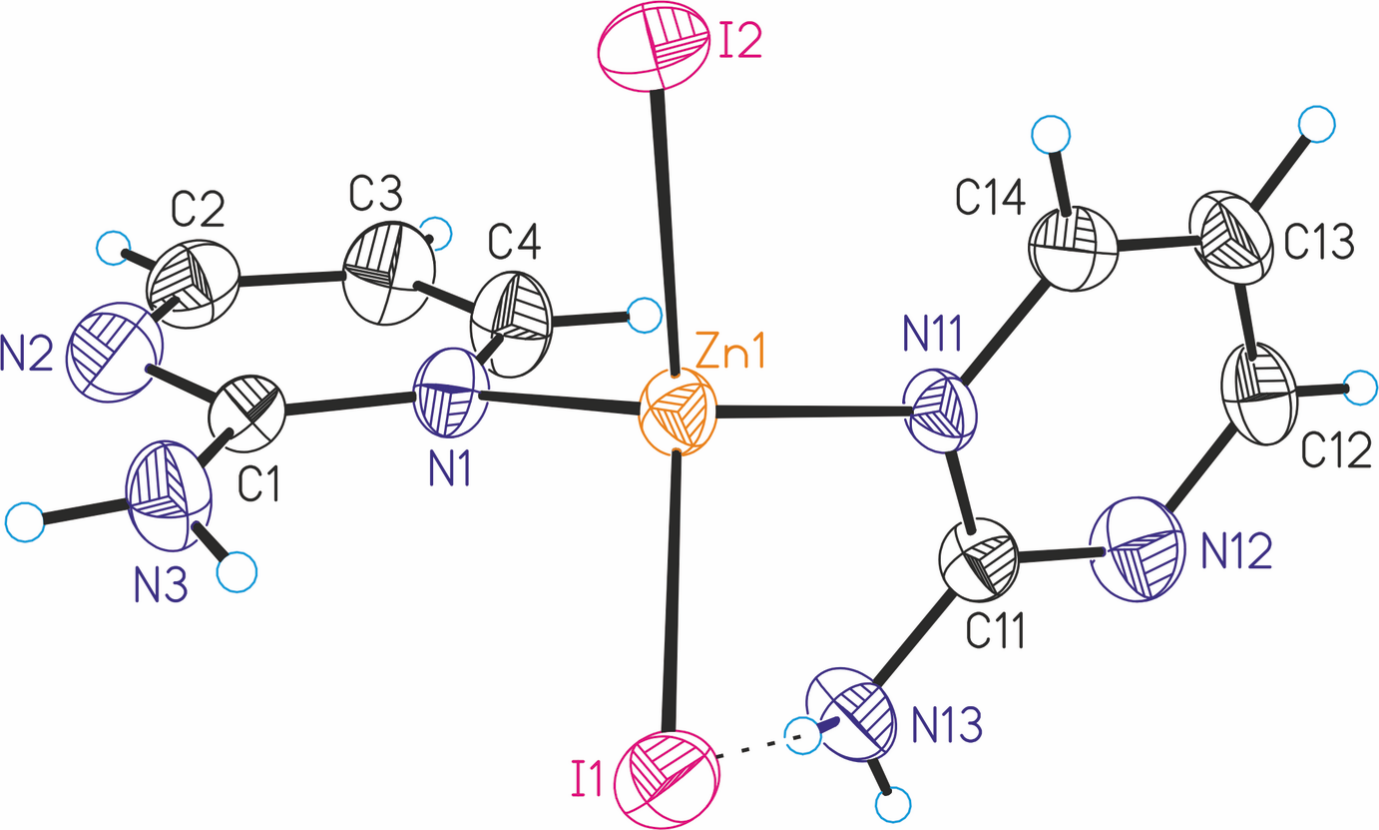
The mol­ecular structure of **2** with displacement ellipsoids drawn at the 50% probability level and the intra­molecular N—H⋯I hydrogen bonding shown as a dashed line.

The iodide compound ZnI_2_(C_4_H_5_N_3_)_2_ (**2**) is not isotypic to any of the zinc(II) halide compounds known in the literature and also not to **1**. Its asymmetric unit is built up of one Zn^II^ cation as well as two crystallographically independent iodide anions and 2-amino­pyrimidine ligands that are located in general positions (Fig. 2 ▸). As in **1**, the Zn^II^ cations are fourfold coordinated by two iodide anions and two 2-amino­pyrimidine ligands within slightly distorted tetra­hedra (Fig. 2 ▸ and Table 2 ▸).

## 3. Supra­molecular features

**Figure 3:**
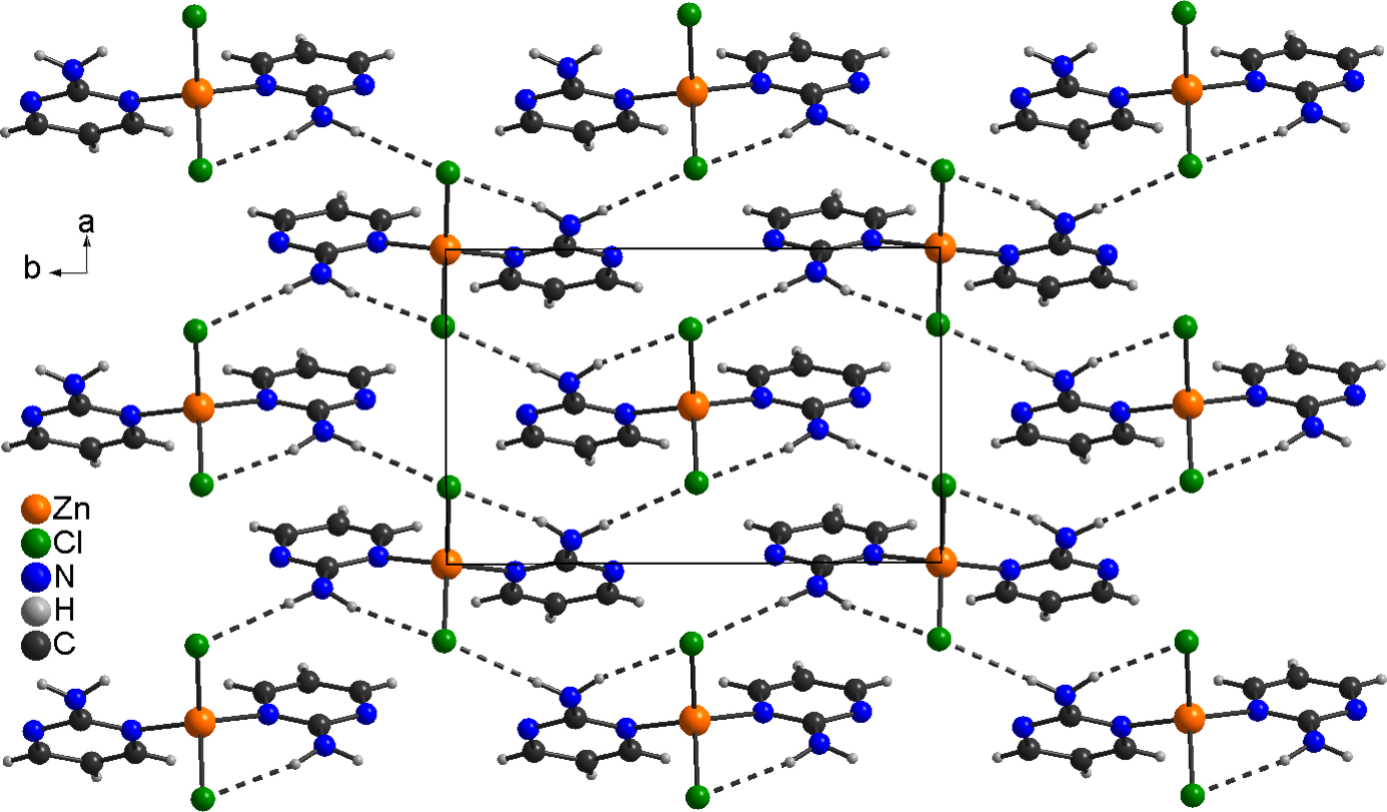
Crystal structure of **1** with a view onto the layers formed by inter­molecular N—H⋯Cl hydrogen bonds (shown as dashed lines).

**Figure 4:**
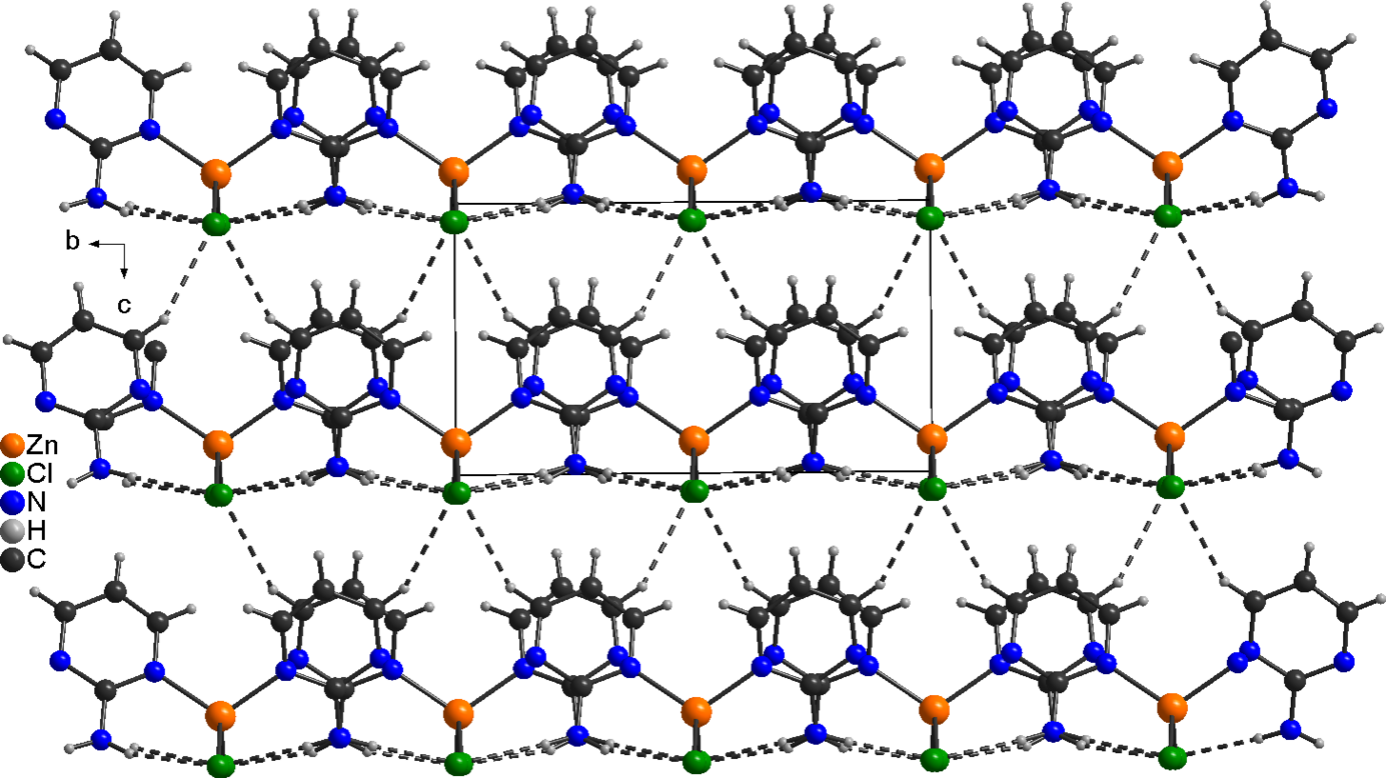
Crystal structure of **1** with a view along the *a*-axis direction showing the non-centrosymmetry of this structure. Inter­molecular hydrogen bonds are shown as dashed lines.

In the extended structure of **1**, the discrete complexes are linked *via* N—H⋯Cl hydrogen bonds between the amino H atom and the chloride anions (Fig. 3 ▸). One of these H atoms acts as donor for an intra­molecular, the second for an inter­molecular hydrogen bond. The H⋯Cl distances are relatively short and the N—H⋯Cl angles close to linear indicate strong hydrogen bonding (Table 3 ▸). In this way, layers are formed,that lie parallel to the *ab* plane with the hydrogen bonds pointed in the direction of the crystallographic *b* axis (Fig. 3 ▸). These layers are further linked by C—H⋯Cl hydrogen bonds between one of the H atoms of the six-membered rings and the chloride anions (Fig. 4 ▸ and Table 3 ▸). From Fig. 4 ▸ it may be seen that all the pyrimidine rings point in the crystallographic *c*-axis direction, clearly proving the non-centrosymmetry of this structure (Fig. 4 ▸).

**Figure 5:**
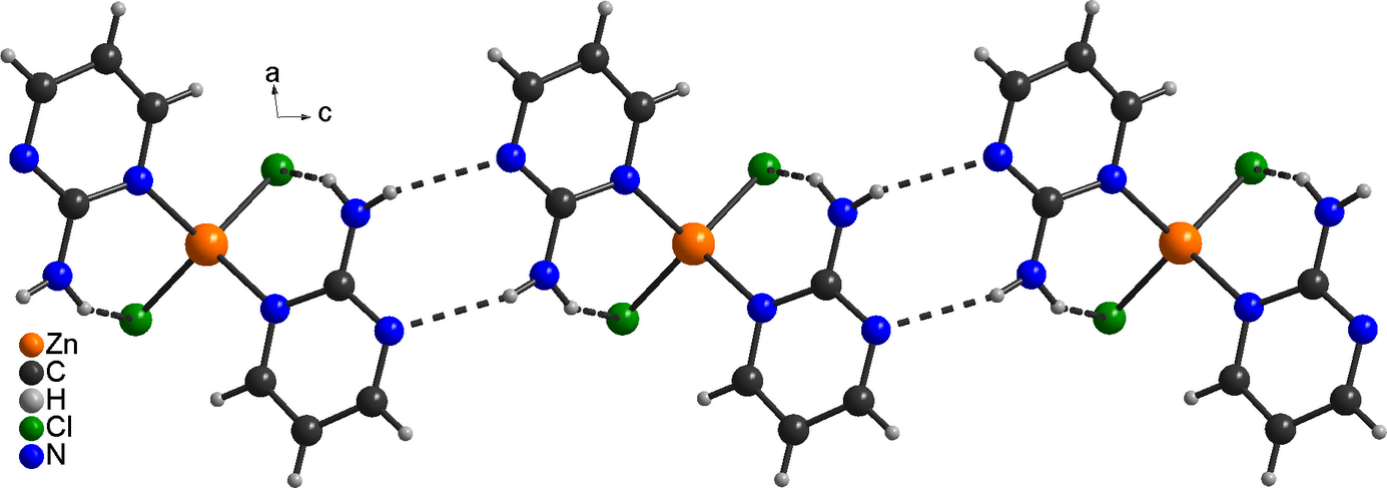
Crystal structure of the second polymorph of **1** already reported in the literature (Lin & Zeng, 2007 ▸) with inter­molecular N—H⋯N and N—H⋯Cl hydrogen bonds shown as dashed lines.

The structure of **1** is completely different from that of the other polymorph already reported in the literature. In this modification, each complex is linked to neighbouring complexes by centrosymmetric pairs of N—H⋯N hydrogen bonds (Fig. 5 ▸). The second N—H H atom is only involved in intra­molecular N—H⋯Cl hydrogen bonding but in contrast to the form presented here, the N—H⋯N and N—H⋯Cl angles are far from linear, surprisingly indicating weaker inter­actions. Finally, in contrast to the title polymorph, in the known form the complexes are linked into chains that propagate in the *c*-axis direction (Fig. 5 ▸).

**Figure 6:**
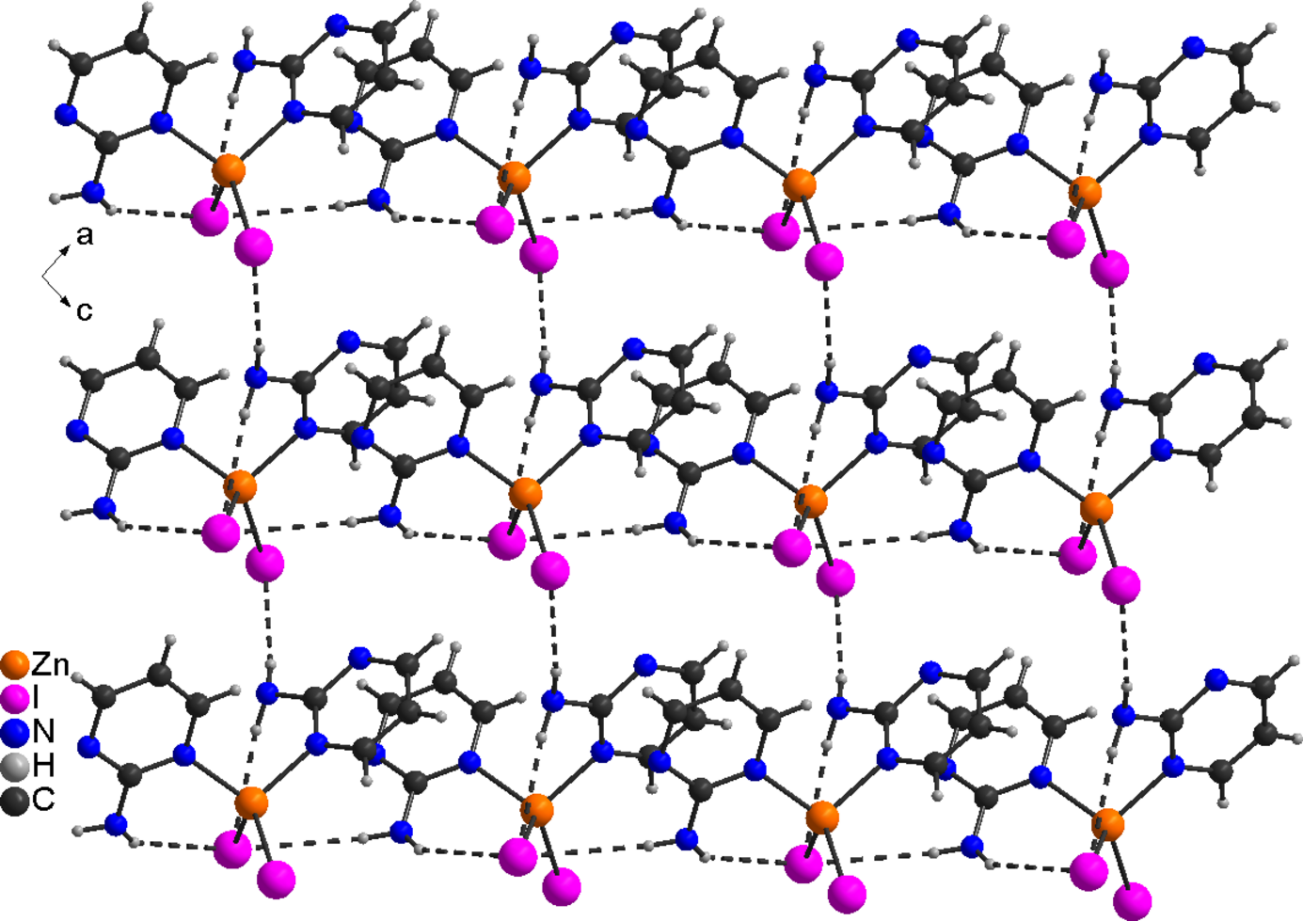
Crystal structure of **2** along the *b*-axis direction with a view onto the layers and inter­molecular N—H⋯I hydrogen bonds shown as dashed lines.

**Figure 7:**
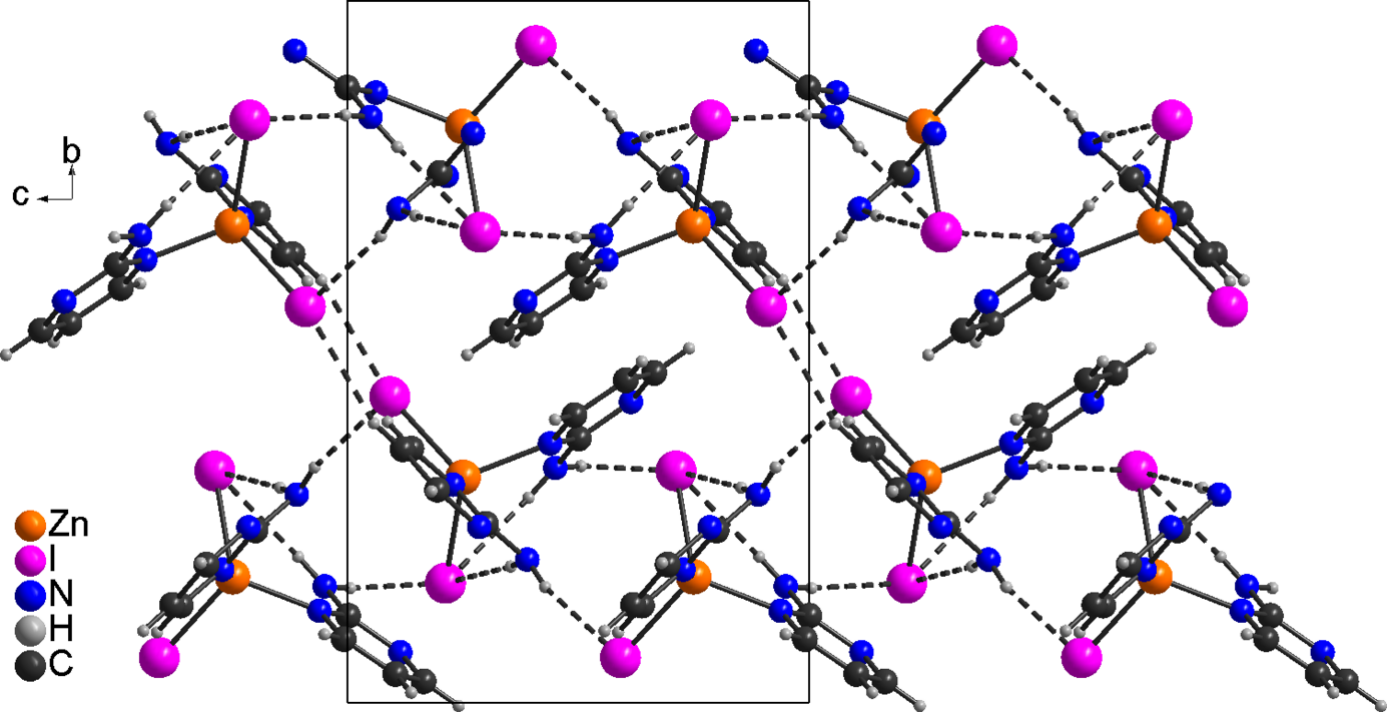
Crystal structure of **2** with a view along the *a*-axis direction and inter­molecular hydrogen bonds shown as dashed lines.

In the iodide compound **2**, the discrete complexes are linked by inter­molecular N—H⋯I hydrogen bonds into layers that in this compound are parallel to the *bc* plane (Fig. 6 ▸). As in compound **1**, one amine H atom is involved in an intra­molecular hydrogen bond, whereas the second H atom shows inter­molecular hydrogen bonding. Bond lengths and angles also indicate a significant inter­action (Table 4 ▸). These layers are further linked by pairs of C—H⋯I inter­actions between the iodide anions and the H atoms of the pyrimidine rings (Fig. 7 ▸).

## 4. Database survey

A search in the CSD (Version 5.43, 2025; Groom *et al.*, 2016 ▸) using CONQUEST (Bruno *et al.*, 2002 ▸) revealed that two Zn halide coordination compounds with the composition ZnCl_2_(C_4_H_5_N_3_)_2_ (Lin & Zeng, 2007 ▸) and ZnBr_2_(C_4_H_5_N_3_)_2_ (Qu *et al.*, 2008 ▸) are already reported (see above). There are additional Zn compounds with different anions that are related to the title compounds, including (Zn(NCS)_2_(C_4_H_5_N_3_)_2_ (Jin *et al.*, 2010 ▸), (Zn(NO_3_)_2_(C_4_H_5_N_3_)_2_(H_2_O) (Gao & Ng, 2010 ▸) and (Zn(C_11_H_5_O_2_F_3_)_2_(C_4_H_5_N_3_)_2_ (Perdih, 2016 ▸) that also consists of discrete complexes. There is also a compound with mixed hydroxide nitrate anions that forms a polymeric structure (Kang *et al.*, 2011 ▸).

Finally, it is noted that some compounds are reported in which the 2-amino­pyrimidine ligands are protonated, but none of them contain zinc(II) as cation. These include, for example, 2-amino-1,3-di­hydro­pyrimidiniumtetra­bromo­copper(II) (Pon *et al.*, 1997 ▸), 2-amino­pyrimidiniumtetra­bromo­cobalt(II) mono­hydrate (Masaki *et al.*, 2002 ▸), 2-amino-1,3-di­hydro­pyrimidin­ium­tetra­aqua­dibromo­manganese(II) dibromide (Lee *et al.*, 2003 ▸) and 2-amino-1,3-di­hydro­pyrimidiniumtetra­aqua­di­bromo­nickel(II) dibromide (Masaki *et al.*, 2002 ▸). In three additional compounds, the 2-amino­pyridine ligand is also protonated and act as counter-cation for Mo and W cluster compounds (Chen *et al.*, 2015 ▸; Xiao *et al.*, 2018 ▸).

## 5. Synthesis and crystallization

Zinc chloride and zinc iodide as well as 2-amino­pyrimidine were purchased from Sigma-Aldrich.

To prepare **1**, 0.500 mmol (68.1 mg) of zinc chloride and 1.00 mmol (95.1 mg) of 2-amino­pyrimidine were reacted in a solvent mixture of 1 ml of tri­chloro­methane and 1 ml of methanol. Within 3 d, crystals suitable for single crystal X-ray diffraction were obtained in the form of colorless blocks.

Compound **2** was prepared by reacting 0.500 mmol (159.6 mg) of zinc iodide and 1.00 mmol (95.1 mg) of 2-amino­pyrimidine in a solvent mixture of 3 ml of tri­chloro­methane and 3 ml of methanol. Within 3 d, colorless blocks suitable for single crystal X-ray diffraction were obtained.

## 6. Refinement

Crystal data, data collection and structure refinement details are summarized in Table 5 ▸. The C—H hydrogen atoms were positioned with idealized geometry and were refined as riding atoms with *U*_iso_(H) = 1.2*U*_eq_(C). The N—H H atoms were located in difference maps and were refined isotropically with restraints (DFIX).

## Supplementary Material

Crystal structure: contains datablock(s) 1, 2. DOI: 10.1107/S2056989026001878/hb8195sup1.cif

Structure factors: contains datablock(s) 1. DOI: 10.1107/S2056989026001878/hb81951sup2.hkl

Structure factors: contains datablock(s) 2. DOI: 10.1107/S2056989026001878/hb81952sup3.hkl

CCDC references: 2531977, 2531976

Additional supporting information:  crystallographic information; 3D view; checkCIF report

## Figures and Tables

**Table 1:** Selected geometric parameters (Å, °) for **1**[Chem scheme1]

Zn1—N1	2.028 (3)	Zn1—Cl1	2.2613 (10)
			
N1^i^—Zn1—N1	111.5 (2)	N1—Zn1—Cl1	111.64 (9)
N1—Zn1—Cl1^i^	105.22 (9)	Cl1^i^—Zn1—Cl1	111.79 (6)

Symmetry code: (i) 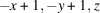.

**Table 2:** Selected geometric parameters (Å, °) for **2**[Chem scheme1]

Zn1—N11	2.040 (3)	Zn1—I2	2.5686 (6)
Zn1—N1	2.052 (4)	Zn1—I1	2.5948 (6)
			
N11—Zn1—N1	103.25 (14)	N11—Zn1—I1	109.47 (10)
N11—Zn1—I2	110.46 (10)	N1—Zn1—I1	114.82 (10)
N1—Zn1—I2	107.01 (10)	I2—Zn1—I1	111.50 (2)

**Table 3:** Hydrogen-bond geometry (Å, °) for **1**[Chem scheme1]

*D*—H⋯*A*	*D*—H	H⋯*A*	*D*⋯*A*	*D*—H⋯*A*
N3—H3*A*⋯Cl1^ii^	0.91 (2)	2.52 (3)	3.431 (4)	177 (5)
N3—H3*B*⋯Cl1	0.92 (2)	2.43 (3)	3.311 (4)	162 (4)
C2—H2⋯Cl1^iii^	0.94	2.92	3.772 (5)	152

Symmetry codes: (ii) 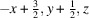; (iii) 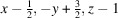.

**Table 4:** Hydrogen-bond geometry (Å, °) for **2**[Chem scheme1]

*D*—H⋯*A*	*D*—H	H⋯*A*	*D*⋯*A*	*D*—H⋯*A*
N3—H3*A*⋯I1	0.88 (2)	2.96 (3)	3.742 (4)	150 (4)
N3—H3*B*⋯I1^i^	0.89 (2)	3.03 (2)	3.922 (4)	173 (5)
C2—H2⋯I2^ii^	0.94	3.31	3.982 (5)	130
C4—H4⋯N11	0.94	2.67	3.275 (6)	123
N13—H13*A*⋯I1	0.90	2.84	3.738 (4)	173
N13—H13*B*⋯I2^iii^	0.90	2.81	3.693 (4)	169
C13—H13⋯I2^iv^	0.94	3.10	4.001 (5)	162
C14—H14⋯I2	0.94	3.14	3.815 (5)	130

Symmetry codes: (i) 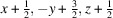; (ii) 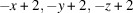; (iii) 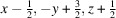; (iv) 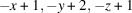.

**Table 5:** Experimental details

	**1**	**2**
Crystal data		
Chemical formula	[ZnCl_2_(C_4_H_5_N_3_)_2_]	[ZnI_2_(C_4_H_5_N_3_)_2_]
*M*_r_	326.49	509.39
Crystal system, space group	Orthorhombic, *Pba*2	Monoclinic, *P*2_1_/*n*
Temperature (K)	220	220
*a*, *b*, *c* (Å)	7.6628 (7), 12.0590 (8), 6.8735 (4)	9.5469 (6), 15.3764 (13), 10.1518 (6)
α, β, γ (°)	90, 90, 90	90, 95.884 (7), 90
*V* (Å^3^)	635.15 (8)	1482.40 (18)
*Z*	2	4
Radiation type	Mo *K*α	Mo *K*α
μ (mm^−1^)	2.34	5.81
Crystal size (mm)	0.18 × 0.16 × 0.14	0.16 × 0.10 × 0.06

Data collection		
Diffractometer	Stoe *IPDS1*	Stoe *IPDS1*
Absorption correction	Numerical (*X-RED* and *X-SHAPE*; Stoe, 2008 ▸)	Numerical (*X-RED* and *X-SHAPE*; Stoe, 2008 ▸)
*T*_min_, *T*_max_	0.636, 0.842	0.296, 0.471
No. of measured, independent and observed [*I* > 2σ(*I*)] reflections	5690, 1490, 1251	14800, 3491, 2946
*R*_int_	0.045	0.091
(sin θ/λ)_max_ (Å^−1^)	0.660	0.661

Refinement		
*R*[*F*^2^ > 2σ(*F*^2^)], *wR*(*F*^2^), *S*	0.032, 0.080, 1.00	0.036, 0.094, 1.03
No. of reflections	1490	3491
No. of parameters	87	163
No. of restraints	3	2
H-atom treatment	H atoms treated by a mixture of independent and constrained refinement	H atoms treated by a mixture of independent and constrained refinement
Δρ_max_, Δρ_min_ (e Å^−3^)	0.49, −0.43	1.17, −1.28
Absolute structure	Flack *x* determined using 505 quotients [(*I*^+^)−(*I*^−^)]/[(*I*^+^)+(*I*^−^)] (Parsons *et al.*, 2013 ▸)	–
Absolute structure parameter	0.007 (13)	–

Computer programs: *X-AREA* (Stoe, 2008 ▸), *SHELXT* (Sheldrick, 2015*a* ▸), *SHELXL* (Sheldrick, 2015*b* ▸), *DIAMOND* (Brandenburg, 1999 ▸) and *XP* in *SHELXTL-PC* (Sheldrick, 2008 ▸) and *publCIF* (Westrip, 2010 ▸).

## supplementary crystallographic information

### Bis(2-aminopyrimidine-κN1)dichloridozinc(II) (1) Crystal data

**Table:**

[ZnCl_2_(C_4_H_5_N_3_)_2_]	*D*_x_ = 1.707 Mg m^−^^3^
*M**_r_* = 326.49	Mo *K*α radiation, λ = 0.71073 Å
Orthorhombic, *P**b**a*2	Cell parameters from 6615 reflections
*a* = 7.6628 (7) Å	θ = 3.0–28.0°
*b* = 12.0590 (8) Å	µ = 2.34 mm^−^^1^
*c* = 6.8735 (4) Å	*T* = 220 K
*V* = 635.15 (8) Å^3^	Block, colorless
*Z* = 2	0.18 × 0.16 × 0.14 mm
*F*(000) = 328	

### Bis(2-aminopyrimidine-κN1)dichloridozinc(II) (1) Data collection

**Table:**

Stoe IPDS-1 diffractometer	1251 reflections with *I* > 2σ(*I*)
Phi scans	*R*_int_ = 0.045
Absorption correction: numerical (X-Red and X-Shape; Stoe, 2008)	θ_max_ = 28.0°, θ_min_ = 3.2°
*T*_min_ = 0.636, *T*_max_ = 0.842	*h* = −10→10
5690 measured reflections	*k* = −15→15
1490 independent reflections	*l* = −8→9

### Bis(2-aminopyrimidine-κN1)dichloridozinc(II) (1) Refinement

**Table:**

Refinement on *F*^2^	H atoms treated by a mixture of independent and constrained refinement
Least-squares matrix: full	*w* = 1/[σ^2^(*F*_o_^2^) + (0.0537*P*)^2^] where *P* = (*F*_o_^2^ + 2*F*_c_^2^)/3
*R*[*F*^2^ > 2σ(*F*^2^)] = 0.032	(Δ/σ)_max_ < 0.001
*wR*(*F*^2^) = 0.080	Δρ_max_ = 0.49 e Å^−^^3^
*S* = 1.00	Δρ_min_ = −0.43 e Å^−^^3^
1490 reflections	Extinction correction: SHELXL-2016/6 (Sheldrick 2015b), Fc^*^=kFc[1+0.001xFc^2^λ^3^/sin(2θ)]^-1/4^
87 parameters	Extinction coefficient: 0.038 (7)
3 restraints	Absolute structure: Flack *x* determined using 505 quotients [(*I*^+^)-(*I*^-^)]/[(*I*^+^)+(*I*^-^)] (Parsons *et al.*, 2013)
Primary atom site location: dual	Absolute structure parameter: 0.007 (13)
Hydrogen site location: mixed	

### Bis(2-aminopyrimidine-κN1)dichloridozinc(II) (1) Special details

**Table:**

Geometry. All esds (except the esd in the dihedral angle between two l.s. planes) are estimated using the full covariance matrix. The cell esds are taken into account individually in the estimation of esds in distances, angles and torsion angles; correlations between esds in cell parameters are only used when they are defined by crystal symmetry. An approximate (isotropic) treatment of cell esds is used for estimating esds involving l.s. planes.

### Bis(2-aminopyrimidine-κN1)dichloridozinc(II) (1) Fractional atomic coordinates and isotropic or equivalent isotropic displacement parameters (Å^2^)

**Table:**

	*x*	*y*	*z*	*U*_iso_*/*U*_eq_	
Zn1	0.500000	0.500000	0.88265 (9)	0.0219 (2)	
Cl1	0.74418 (15)	0.50555 (6)	1.06713 (13)	0.0302 (3)	
N1	0.4752 (4)	0.6381 (3)	0.7166 (5)	0.0222 (7)	
N2	0.4748 (6)	0.8362 (4)	0.6738 (8)	0.0446 (12)	
N3	0.5753 (6)	0.7516 (3)	0.9660 (7)	0.0349 (9)	
H3A	0.623 (6)	0.819 (3)	0.998 (8)	0.035 (12)*	
H3B	0.634 (5)	0.692 (3)	1.018 (7)	0.029 (12)*	
C1	0.5104 (5)	0.7413 (4)	0.7842 (6)	0.0232 (8)	
C2	0.4046 (7)	0.8250 (4)	0.4924 (8)	0.0388 (11)	
H2	0.378192	0.888020	0.417373	0.047*	
C3	0.3723 (6)	0.7190 (4)	0.4194 (6)	0.0372 (11)	
H3	0.328392	0.709206	0.292874	0.045*	
C4	0.4057 (6)	0.6306 (3)	0.5345 (7)	0.0304 (9)	
H4	0.379338	0.559649	0.486091	0.036*	

### Bis(2-aminopyrimidine-κN1)dichloridozinc(II) (1) Atomic displacement parameters (Å^2^)

**Table:**

	*U*^11^	*U*^22^	*U*^33^	*U*^12^	*U*^13^	*U*^23^
Zn1	0.0307 (3)	0.0128 (3)	0.0223 (3)	−0.0020 (2)	0.000	0.000
Cl1	0.0370 (6)	0.0213 (5)	0.0322 (6)	0.0024 (4)	−0.0098 (4)	−0.0014 (5)
N1	0.0293 (18)	0.0158 (14)	0.0215 (17)	−0.0022 (11)	−0.0010 (13)	0.0017 (13)
N2	0.058 (3)	0.029 (2)	0.047 (3)	0.0026 (17)	0.004 (2)	0.008 (2)
N3	0.058 (2)	0.0152 (14)	0.032 (2)	−0.0020 (19)	−0.011 (2)	−0.0034 (14)
C1	0.0279 (19)	0.0170 (18)	0.025 (2)	−0.0020 (15)	0.0059 (17)	0.0000 (16)
C2	0.055 (3)	0.030 (2)	0.031 (2)	0.008 (2)	−0.002 (2)	0.0150 (19)
C3	0.046 (2)	0.042 (2)	0.024 (3)	0.005 (2)	−0.0037 (18)	0.0048 (18)
C4	0.035 (2)	0.030 (2)	0.026 (2)	−0.0036 (16)	−0.0062 (18)	−0.0002 (16)

### Bis(2-aminopyrimidine-κN1)dichloridozinc(II) (1) Geometric parameters (Å, º)

**Table:**

Zn1—N1^i^	2.028 (3)	N3—C1	1.351 (6)
Zn1—N1	2.028 (3)	N3—H3A	0.91 (2)
Zn1—Cl1^i^	2.2612 (10)	N3—H3B	0.92 (2)
Zn1—Cl1	2.2613 (10)	C2—C3	1.396 (7)
N1—C1	1.355 (6)	C2—H2	0.9400
N1—C4	1.363 (6)	C3—C4	1.352 (6)
N2—C2	1.365 (7)	C3—H3	0.9400
N2—C1	1.401 (6)	C4—H4	0.9400
			
N1^i^—Zn1—N1	111.5 (2)	N3—C1—N1	118.4 (4)
N1^i^—Zn1—Cl1^i^	111.64 (9)	N3—C1—N2	119.8 (4)
N1—Zn1—Cl1^i^	105.22 (9)	N1—C1—N2	121.7 (4)
N1^i^—Zn1—Cl1	105.22 (9)	N2—C2—C3	119.3 (4)
N1—Zn1—Cl1	111.64 (9)	N2—C2—H2	120.4
Cl1^i^—Zn1—Cl1	111.79 (6)	C3—C2—H2	120.4
C1—N1—C4	117.0 (3)	C4—C3—C2	118.6 (4)
C1—N1—Zn1	122.8 (3)	C4—C3—H3	120.7
C4—N1—Zn1	119.9 (3)	C2—C3—H3	120.7
C2—N2—C1	119.4 (4)	C3—C4—N1	124.0 (4)
C1—N3—H3A	117 (3)	C3—C4—H4	118.0
C1—N3—H3B	118 (3)	N1—C4—H4	118.0
H3A—N3—H3B	114 (4)		

Symmetry code: (i) −*x*+1, −*y*+1, *z*.

### Bis(2-aminopyrimidine-κN1)dichloridozinc(II) (1) Hydrogen-bond geometry (Å, º)

**Table:**

*D*—H···*A*	*D*—H	H···*A*	*D*···*A*	*D*—H···*A*
N3—H3*A*···Cl1^ii^	0.91 (2)	2.52 (3)	3.431 (4)	177 (5)
N3—H3*B*···Cl1	0.92 (2)	2.43 (3)	3.311 (4)	162 (4)
C2—H2···Cl1^iii^	0.94	2.92	3.772 (5)	152

Symmetry codes: (ii) −*x*+3/2, *y*+1/2, *z*; (iii) *x*−1/2, −*y*+3/2, *z*−1.

### Bis(2-aminopyrimidine-κN1)diiodidozinc(II) (2) Crystal data

**Table:**

[ZnI_2_(C_4_H_5_N_3_)_2_]	*F*(000) = 944
*M**_r_* = 509.39	*D*_x_ = 2.282 Mg m^−^^3^
Monoclinic, *P*2_1_/*n*	Mo *K*α radiation, λ = 0.71073 Å
*a* = 9.5469 (6) Å	Cell parameters from 8000 reflections
*b* = 15.3764 (13) Å	θ = 10.4–25.7°
*c* = 10.1518 (6) Å	µ = 5.81 mm^−^^1^
β = 95.884 (7)°	*T* = 220 K
*V* = 1482.40 (18) Å^3^	Block, colorless
*Z* = 4	0.16 × 0.10 × 0.06 mm

### Bis(2-aminopyrimidine-κN1)diiodidozinc(II) (2) Data collection

**Table:**

Stoe IPDS-1 diffractometer	2946 reflections with *I* > 2σ(*I*)
Phi scans	*R*_int_ = 0.091
Absorption correction: numerical (X-Red and X-Shape; Stoe, 2008)	θ_max_ = 28.0°, θ_min_ = 2.4°
*T*_min_ = 0.296, *T*_max_ = 0.471	*h* = −12→12
14800 measured reflections	*k* = −20→20
3491 independent reflections	*l* = −13→13

### Bis(2-aminopyrimidine-κN1)diiodidozinc(II) (2) Refinement

**Table:**

Refinement on *F*^2^	Hydrogen site location: mixed
Least-squares matrix: full	H atoms treated by a mixture of independent and constrained refinement
*R*[*F*^2^ > 2σ(*F*^2^)] = 0.036	*w* = 1/[σ^2^(*F*_o_^2^) + (0.0549*P*)^2^] where *P* = (*F*_o_^2^ + 2*F*_c_^2^)/3
*wR*(*F*^2^) = 0.094	(Δ/σ)_max_ = 0.001
*S* = 1.03	Δρ_max_ = 1.17 e Å^−^^3^
3491 reflections	Δρ_min_ = −1.28 e Å^−^^3^
163 parameters	Extinction correction: SHELXL-2016/6 (Sheldrick 2015b), Fc^*^=kFc[1+0.001xFc^2^λ^3^/sin(2θ)]^-1/4^
2 restraints	Extinction coefficient: 0.0054 (4)
Primary atom site location: dual	

### Bis(2-aminopyrimidine-κN1)diiodidozinc(II) (2) Special details

**Table:**

Geometry. All esds (except the esd in the dihedral angle between two l.s. planes) are estimated using the full covariance matrix. The cell esds are taken into account individually in the estimation of esds in distances, angles and torsion angles; correlations between esds in cell parameters are only used when they are defined by crystal symmetry. An approximate (isotropic) treatment of cell esds is used for estimating esds involving l.s. planes.

### Bis(2-aminopyrimidine-κN1)diiodidozinc(II) (2) Fractional atomic coordinates and isotropic or equivalent isotropic displacement parameters (Å^2^)

**Table:**

	*x*	*y*	*z*	*U*_iso_*/*U*_eq_	
Zn1	0.75540 (4)	0.82087 (3)	0.75052 (5)	0.02767 (14)	
I1	0.86634 (3)	0.67008 (2)	0.71225 (3)	0.03788 (12)	
I2	0.83722 (3)	0.93595 (2)	0.59175 (3)	0.03509 (12)	
N1	0.7972 (4)	0.8692 (2)	0.9389 (3)	0.0299 (7)	
N2	0.9550 (5)	0.9284 (3)	1.1144 (5)	0.0474 (10)	
N3	1.0345 (4)	0.8344 (3)	0.9503 (5)	0.0391 (9)	
H3A	1.031 (5)	0.792 (2)	0.892 (4)	0.033 (13)*	
H3B	1.113 (4)	0.837 (4)	1.006 (5)	0.049 (16)*	
C1	0.9279 (4)	0.8763 (3)	1.0011 (4)	0.0293 (8)	
C2	0.8455 (5)	0.9696 (3)	1.1649 (5)	0.0407 (10)	
H2	0.861854	1.004769	1.240675	0.049*	
C3	0.7096 (5)	0.9592 (4)	1.1032 (6)	0.0460 (11)	
H3	0.632564	0.985892	1.137634	0.055*	
C4	0.6905 (5)	0.9095 (3)	0.9917 (5)	0.0403 (10)	
H4	0.598736	0.902899	0.949621	0.048*	
N11	0.5413 (3)	0.8105 (2)	0.7281 (4)	0.0286 (7)	
N12	0.3223 (4)	0.7503 (3)	0.7851 (5)	0.0480 (10)	
N13	0.5463 (4)	0.7037 (3)	0.8897 (4)	0.0405 (9)	
H13A	0.627117	0.693806	0.853657	0.061*	
H13B	0.493126	0.664756	0.928267	0.061*	
C11	0.4698 (4)	0.7547 (3)	0.8001 (4)	0.0296 (8)	
C12	0.2495 (4)	0.8033 (3)	0.6940 (5)	0.0370 (10)	
H12	0.150638	0.801076	0.681757	0.044*	
C13	0.3224 (5)	0.8608 (3)	0.6193 (5)	0.0397 (10)	
H13	0.273687	0.898093	0.557058	0.048*	
C14	0.4642 (5)	0.8618 (3)	0.6384 (5)	0.0371 (9)	
H14	0.512675	0.899989	0.586882	0.044*	

### Bis(2-aminopyrimidine-κN1)diiodidozinc(II) (2) Atomic displacement parameters (Å^2^)

**Table:**

	*U*^11^	*U*^22^	*U*^33^	*U*^12^	*U*^13^	*U*^23^
Zn1	0.0211 (2)	0.0315 (2)	0.0306 (3)	−0.00168 (16)	0.00331 (19)	0.00062 (17)
I1	0.02970 (17)	0.03171 (17)	0.0529 (2)	0.00108 (10)	0.00748 (13)	−0.00385 (11)
I2	0.03910 (19)	0.02980 (17)	0.03796 (19)	−0.00264 (10)	0.01170 (13)	0.00272 (10)
N1	0.0257 (16)	0.0353 (18)	0.0290 (18)	−0.0031 (13)	0.0035 (14)	−0.0019 (13)
N2	0.048 (2)	0.045 (2)	0.049 (3)	−0.0071 (18)	0.003 (2)	−0.0010 (18)
N3	0.0285 (19)	0.046 (2)	0.042 (2)	0.0032 (15)	−0.0006 (17)	−0.0062 (17)
C1	0.0274 (19)	0.0267 (19)	0.033 (2)	−0.0041 (14)	0.0008 (16)	0.0029 (15)
C2	0.049 (3)	0.036 (2)	0.037 (2)	−0.0031 (19)	0.006 (2)	−0.0065 (19)
C3	0.042 (3)	0.049 (3)	0.048 (3)	0.003 (2)	0.009 (2)	−0.013 (2)
C4	0.027 (2)	0.049 (3)	0.045 (3)	0.0028 (18)	0.0058 (19)	−0.012 (2)
N11	0.0200 (15)	0.0340 (17)	0.0314 (19)	−0.0005 (12)	0.0000 (14)	0.0044 (13)
N12	0.034 (2)	0.055 (3)	0.055 (3)	−0.0010 (17)	0.0069 (19)	0.000 (2)
N13	0.0268 (18)	0.050 (2)	0.044 (2)	−0.0027 (15)	0.0034 (16)	0.0186 (18)
C11	0.0246 (19)	0.034 (2)	0.031 (2)	−0.0015 (14)	0.0058 (16)	0.0016 (15)
C12	0.0203 (19)	0.052 (3)	0.038 (3)	0.0063 (17)	0.0002 (17)	−0.004 (2)
C13	0.034 (2)	0.043 (3)	0.040 (3)	0.0090 (18)	−0.0064 (19)	0.0030 (19)
C14	0.034 (2)	0.034 (2)	0.043 (3)	−0.0013 (17)	−0.0001 (19)	0.0050 (18)

### Bis(2-aminopyrimidine-κN1)diiodidozinc(II) (2) Geometric parameters (Å, º)

**Table:**

Zn1—N11	2.040 (3)	C3—H3	0.9400
Zn1—N1	2.052 (4)	C4—H4	0.9400
Zn1—I2	2.5686 (6)	N11—C11	1.356 (5)
Zn1—I1	2.5948 (6)	N11—C14	1.361 (6)
N1—C1	1.344 (5)	N12—C12	1.368 (6)
N1—C4	1.349 (6)	N12—C11	1.403 (6)
N2—C2	1.367 (7)	N13—C11	1.356 (6)
N2—C1	1.403 (6)	N13—H13A	0.9000
N3—C1	1.351 (6)	N13—H13B	0.9001
N3—H3A	0.876 (19)	C12—C13	1.396 (7)
N3—H3B	0.89 (2)	C12—H12	0.9400
C2—C3	1.391 (7)	C13—C14	1.348 (6)
C2—H2	0.9400	C13—H13	0.9400
C3—C4	1.363 (7)	C14—H14	0.9400
			
N11—Zn1—N1	103.25 (14)	N1—C4—C3	123.1 (4)
N11—Zn1—I2	110.46 (10)	N1—C4—H4	118.5
N1—Zn1—I2	107.01 (10)	C3—C4—H4	118.5
N11—Zn1—I1	109.47 (10)	C11—N11—C14	117.3 (4)
N1—Zn1—I1	114.82 (10)	C11—N11—Zn1	122.9 (3)
I2—Zn1—I1	111.50 (2)	C14—N11—Zn1	119.8 (3)
C1—N1—C4	118.6 (4)	C12—N12—C11	118.6 (4)
C1—N1—Zn1	123.4 (3)	C11—N13—H13A	104.5
C4—N1—Zn1	117.0 (3)	C11—N13—H13B	112.8
C2—N2—C1	119.2 (4)	H13A—N13—H13B	127.6
C1—N3—H3A	129 (4)	N11—C11—N13	117.5 (4)
C1—N3—H3B	111 (4)	N11—C11—N12	121.8 (4)
H3A—N3—H3B	115 (5)	N13—C11—N12	120.7 (4)
N1—C1—N3	118.8 (4)	N12—C12—C13	119.9 (4)
N1—C1—N2	121.0 (4)	N12—C12—H12	120.1
N3—C1—N2	120.2 (4)	C13—C12—H12	120.1
N2—C2—C3	119.5 (4)	C14—C13—C12	118.5 (4)
N2—C2—H2	120.3	C14—C13—H13	120.7
C3—C2—H2	120.3	C12—C13—H13	120.7
C4—C3—C2	118.5 (5)	C13—C14—N11	123.8 (4)
C4—C3—H3	120.7	C13—C14—H14	118.1
C2—C3—H3	120.7	N11—C14—H14	118.1

### Bis(2-aminopyrimidine-κN1)diiodidozinc(II) (2) Hydrogen-bond geometry (Å, º)

**Table:**

*D*—H···*A*	*D*—H	H···*A*	*D*···*A*	*D*—H···*A*
N3—H3*A*···I1	0.88 (2)	2.96 (3)	3.742 (4)	150 (4)
N3—H3*B*···I1^i^	0.89 (2)	3.03 (2)	3.922 (4)	173 (5)
C2—H2···I2^ii^	0.94	3.31	3.982 (5)	130
C4—H4···N11	0.94	2.67	3.275 (6)	123
N13—H13*A*···I1	0.90	2.84	3.738 (4)	173
N13—H13*B*···I2^iii^	0.90	2.81	3.693 (4)	169
C13—H13···I2^iv^	0.94	3.10	4.001 (5)	162
C14—H14···I2	0.94	3.14	3.815 (5)	130

Symmetry codes: (i) *x*+1/2, −*y*+3/2, *z*+1/2; (ii) −*x*+2, −*y*+2, −*z*+2; (iii) *x*−1/2, −*y*+3/2, *z*+1/2; (iv) −*x*+1, −*y*+2, −*z*+1.
